# Impact of Soybean Meal, Mustard Meal, Rapeseed Meal and Black Cumin on Production Performance, Egg Quality and Gut Microflora of Laying Hens

**DOI:** 10.1002/vms3.70863

**Published:** 2026-03-11

**Authors:** Md Abubakar Siddik, Mst Afroza Khatun, Sweety Rani Mondal, Shoriful Islam, Md. Azizul Haque, Khadiza Akter Brishty, Hemayet Hossain, Md. Mahfujur Rahman

**Affiliations:** ^1^ Department of Dairy and Poultry Science, Faculty of Veterinary and Animal Science Hajee Mohammad Danesh Science and Technology University Dinajpur Bangladesh; ^2^ Department of Zoology (GSSC) University of Dhaka Dhaka Bangladesh; ^3^ Department of Veterinary Science and Animal Husbandry Teesta University Rangpur Bangladesh; ^4^ Department of Medicine; Faculty of Veterinary, Animal and Biomedical Sciences Sylhet Agricultural University Sylhet Bangladesh

**Keywords:** cost‐effectiveness, egg quality, gut microflora, laying hen, poultry nutrition, protein source

## Abstract

**Background and Objectives:**

This study aimed to assess the impact of diverse protein sources (soybean, mustard, rapeseed and black cumin [BC]) as dietary supplements on production performance, egg quality and gut microflora of Shaver Brown 579 commercial layers.

**Methods:**

A randomized design allocated 324 hens over 4 months into six treatments with six replications for each treatment, each replication containing 09 birds. The treatments included T0 (control diet), T1 (basal diet + soybean), T2 (basal diet + mustard), T3 (basal diet + rapeseed), T4 (basal diet + mustard + BC) and T5 (Basal diet + Rapeseed + Black cumin). Production performances were assessed monthly and egg quality characters were assessed at 44th and 52nd weeks.

**Results:**

Hens on diets featuring mustard, rapeseed and BC exhibited significantly higher body weight gain (BWG), without substantial impact on feed intake. The highest final body weight was observed in T5, with no mortality occurrences. No significant differences emerged in egg production, albumen index, shape index, shell breaking strength, shell thickness or shell percentage compared to control groups. However, egg weight showed significance in the third month (*p *< 0.05). Notably, dietary protein sources influenced gut microflora significantly (*p *< 0.05), with T0 having the highest microbial load and T5 the lowest. Egg production cost was the lowest in T4 (7.13 Tk. or $0.067/egg), where mustard oil cake and BC replaced soybean meal (SBM), whereas T1 recorded the highest cost (7.68 Tk. or $0.072/egg) using SBM.

**Conclusion:**

Mustard meal, rapeseed meal and BC are effective SBM substitutes in layer diets without any adverse effect on egg quality or production. These findings highlight the potential of mustard meal, rapeseed meal and BC as quality protein sources in commercial layer nutrition.

## Graphical abstract

The inclusion of novel protein sources such as mustard meal, rapeseed meal and black cumin in layer diets demonstrates promising results without compromising egg quality, improves body weight gain and reduced gut microflora and *Escherichia coli*. Overall, strategically integrating these alternative protein sources shows potential for cost savings.

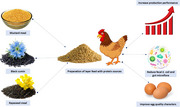

## 1 Introduction

The poultry industry, especially broiler and layer production, plays a crucial role in Bangladesh's economy by generating employment and contributing to food security (Hossain et al. 2025; Rahman et al. 2025). Additionally, it ensures the availability of high‐quality protein, supporting the nutritional needs of the population (Rahman et al. 2024; Tanni et al. 2025). In Bangladesh, approximately 92.25 lakh metric tons of meat and around 2374.97 crore eggs were produced in the fiscal year 2023–2024 (DLS 2024). However, the rising costs of feed ingredients have emerged as a major concern for chicken meat and egg production in Bangladesh (Seidavi et al. 2020). Protein is the second‐largest component in poultry diets, with soybean meal (SBM) being the preferred source for laying hens due to its rich nutrition and amino acid profile. However, protein sources, whether animal‐ or plant‐based, are costlier and less available than energy sources in commercial poultry farming (Toomer et al. 2024). Rising egg consumption and population growth drive competition for SBM between poultry and human food, highlighting the need for sustainable alternatives in layer diets (Pootthachaya et al. 2023). SBM is a key protein source in poultry diets, but its rising cost and limited availability highlight the need for alternatives like mustard, rapeseed and black cumin (BC) meals, which offer sustainable, locally available options. Cottonseed meal (CSM) and rapeseed meal (RM) emerge as significant non‐traditional protein sources in egg production (Gheisari and Ghayor 2014; He et al. 2015). However, challenges arise from conflicting nutritional components (free gossypol and glucosinolate) and the relatively inefficient digestion of essential amino acids (Adeyemo 2008; Gheisari and Ghayor 2014).

Rapeseed meal (30%–40% protein) is rich in lysine, methionine, tryptophan and minerals like calcium, phosphorus and selenium but contains anti‐nutritional factors like glucosinolates and phytic acid (Li et al. 2015). Black cumin (*Nigella sativa*) seeds have 21.07% protein and 39.02% fat and are high in calcium, potassium and magnesium, with oil rich in linoleic (57.71%) and oleic (24.46%) acids (Albakry et al. 2022; M. S. Hossain et al. 2024). Mustard meal (32%–36% protein and 31%–36% oil) is a by‐product of mustard oil extraction, containing methionine, lysine and unsaturated fatty acids like oleic and linoleic acids (Batool et al. 2024; Singh 2023).

Animal proteins are key in poultry diets, whereas plant sources like SBM, cottonseed, sunflower and groundnut cakes are widely used. Poultry rations mainly rely on natural feedstuffs, with occasional amino acid supplementation (Azarnik et al. 2010). Recent studies highlight mustard seed, BC and pawpaw as phytogenic feed additives with proven antibacterial and antihelminthic effects, enhancing broiler productivity (Adegbeye et al. 2020).

High feed costs hinder poultry growth in Bangladesh, reducing animal protein consumption. Using local protein sources like mustard oil cake, til oil cake, BC meal and dried distillers grains with solubles (DDGS) instead of imported fishmeal and SBM can lower costs and boost production without significantly affecting egg yield (Navidshad et al. 2025; Yılmaz et al. 2024). The critical threshold for replacing SBM in poultry feed has been discussed in various studies (Acar et al. 2024; Toomer et al. 2024). For example, SBM is considered the benchmark protein source for poultry feed due to its high digestibility and essential amino acid content. However, exceeding certain replacement levels with alternative protein sources can negatively impact poultry performance, including egg production (Acar et al. 2024; Toomer et al. 2024; Yalçin et al. 2025).

Black cumin (up to 4%) enhances egg production, feed efficiency and yolk colour but may reduce feed intake and body weight at higher levels (Abdollahi et al. 2024; Navidshad et al. 2025). Mustard meal can replace SBM up to 10% without adverse effects, but excessive use may lower egg production and feed intake due to glucosinolates (Yalçin et al. 2025). Rapeseed meal (up to 10%) supports egg production and nutrient digestibility, but higher inclusion may reduce egg weight and production (Konkol et al. 2024).

Alternative proteins like mustard oil cake, blood meal and rapeseed meal reduce reliance on costly SBM and fishmeal. These sources stabilize feed prices and offer a sustainable solution for poultry production. This study aimed to evaluate the effects of alternative protein sources on egg production, quality and gut microflora in laying hens to determine optimal inclusion levels. The findings supported sustainable, cost‐effective poultry feed formulation.

## 2 Materials and Methods

### 2.1 Ethical Statement

This study has undergone rigorous review and received approval from the Animal Ethical Committee of the Institute of Research and Training (IRT) at Hajee Mohammad Danesh Science and Technology University. The approved animal use protocol number is HSTU/IRT/4012.

### 2.2 Preparation of Experimental Site

The experiment spanned from July to December 2022. The experimental site underwent regular cleaning using tap water and was washed every 21 days. Subsequently, all surfaces, including the floor, cages, ceiling and walls, were disinfected with Virocid (6 mL/L) (CID LINES, Belgium, Europe). Each poultry cage/pan was designed to accommodate nine birds in a single compartments.

### 2.3 Experimental Design, Birds Assortment and Diet

In this study, a completely randomized design (CRD) was employed to ensure unbiased allocation of birds across experimental groups. A total of 324 commercial layer hens of the Shaver Brown 579 strain, aged 36 weeks, were randomly assigned to six different treatment groups. Each treatment group was replicated six times (06), with each replication consisting of nine (09) birds. The birds were sourced from local poultry breeders, specifically Provita Breeders Limited (PBL), a subsidiary of Provita Group, Bangladesh. The trial was conducted over a period of 4 months to evaluate the effects of different treatments on the experimental parameters. Experimental birds in T0, T1, T2, T3, T4 and T5 were provided T0 = feed contain Fish analogue protein and SBM as control; T1 = feed contain SBM; T2 = feed contain mustard meal (MM) as protein replacer of SBM; T3 = feed contain rapeseed meal (RM) as protein replacer of SBM; T4 = feed contain MM and BC as protein replacer of SBM and T5 = feed contain RM and BC as protein replacer of SBM. For ration formulation in the trial, raw ingredients were sourced from the local market in Dinajpur, Bangladesh.

The formulated feed comprised a variety of raw ingredients to ensure balanced nutrition for the trial birds, as detailed in Table 1. The primary energy sources included maize and rice polish, whereas SBM, mustard oil cake and rapeseed meal served as protein sources. Soybean oil was incorporated to enhance energy content. Essential additives, such as Pro‐Pak, BC, dicalcium phosphate (DCP), limestone, salt and soda, were included to support overall health and bone development. A vitamin–mineral premix, along with lysine and methionine, was added to meet essential amino acid requirements. To enhance feed quality and safety, a toxin binder, enzyme, Emulex and an anti‐Salmonella agent were incorporated. Additionally, choline chloride was included to support liver function and overall metabolic processes.

**TABLE 1:** Chemical composition of the experimental diets with feed ingredients.

	Dietary treatment					
Feed ingredients (kg)	T0	T1	T2	T3	T4	T5
Maize	58.0	57	57	57	55.5	55
Rice polish	3.5	3	3	3	3.5	3
Soya bean oil	1.5	2	1	2	1	2
Soya bean meal	23	28	—	—	—	—
Pro‐Pak	4	—	6	6	5	4.5
Mustard oil cake	—	—	23	—	22	—
Rapeseed meal	—	—	—	22	—	22
Black cumin	—	—	—	—	3	3.5
DCP	0.65	0.65	0.65	0.65	0.65	0.65
Limestone	9	9	9	9	9	9
Salt	0.35	0.35	0.35	0.35	0.35	0.35
Soda	100	100	100	100	100	100
Vitamin–mineral premix^a^ (g)	250	250	250	250	250	250
Lysine	50	50	50	50	50	50
Methionine	150	150	150	150	150	150
Toxin binder	150	150	150	150	150	150
Enzyme	50	50	50	50	50	50
Emulex	50	50	50	50	50	50
Anti‐Salmonella	150	150	150	150	150	150
Choline chloride	50	50	50	50	50	50
Total	100	100	100	100	100	100
**Chemical composition**						
ME (kcal/kg)	2795.4	2795.5	2795.1	2795.9	2795.7	2795.89
CP (%)	17.82	17.28	17.54	17.62	17.39	17.55
CF (%)	2.945	3.56	4.05	3.93	4.105	4.125
Ca (%)	4.08	3.85	4.32	4.18	3.94	4.06
P (%)	0.57	0.494	0.69	0.484	0.67	0.54
EE (%)	6.47	6.26	5.97	5.55	6.00	6.76
Lysine (%)	0.92	0.89	0.99	0.83	1.04	0.90
Methionine (%)	0.61	0.58	0.61%	0.59	0.63	0.57

Abbreviation: DCP, dicalcium phosphate.

^a^ Added vitamin–mineral premix (Rena‐Layer, Renata Animal Health Ltd)@ 250 g/100 kg which contained: vitamin A: 4800 IU; vitamin D: 960 IU; vitamin E: 9.2 mg; vitamin k_3_: 800 mg; vitamin B_1_: 600 mg; vitamin B_2_: 2 mg; vitamin B_3_: 12 mg; vitamin B_5_: 3.2 mg; vitamin B_6_: 1.8 mg; vitamin B_9_: 2 mg; vitamin B_12_: 0.004 mg; Co: 0.3 mg; Cu: 2.6 mg; Fe: 9.6 mg; I: 0.6 mg; Mn: 19.2 mg; Zn: 16 mg; Se: 0.48 mg; DL‐methionine: 20 mg; l‐lysine: 12 mg. Added lysine 50 g, methionine 150 g, toxin binder 150 g, enzyme 50 g, Emulex 50 g and choline chloride 50 g/100 kg feed.

These ingredients underwent proximate analysis to assess their standard nutritional composition. Mustard meal, rapeseed meal and BC were specifically analysed, and the nutritional composition is provided in Supporting Information File 1. Hand mixed poultry ration was provided to the experimental bird. The ration was formulated to fulfil all nutritional needs as indicated by the 9th revised edition of National Research Council for layer and was classified as the control diet. All the ingredients were taken at proper rate according to their standard composition was shown in Table 1.

### 2.4 Management Tactics, Bio‐Security and Sanitation

Immediately after distribution of layer in the cage, electrolyte and vitamin solutions were provided to drinking water for 4 h. All the treatment groups were offered ad libitum drinking water and feed throughout the experimental trial. Throughout the course of the experiment, the birds were subjected to 16 h of lighting and an 8‐h dark period each day. Visitors were prohibited from entering the experimental housing, and all equipment was kept spick‐and‐span. All care and managements of experimental birds were provided according to the principles and specific guidelines presented in CONSORT research guideline for experimental animal.

#### 2.4.1 Iso‐Caloric Diet Preparation and Feed Ingredient Analysis

The total caloric content of the diet was rigorously maintained at a consistent level across various treatments. Modifications were solely made to the different ingredients used. Initially, the raw ingredients underwent analysis to assess their distinct compositions. Subsequently, the necessary quantities were calculated to create an iso‐caloric diet. The nutrient profiles were evaluated according to the methodology outlined in Aller et al. (2017). The essential nutritional composition of recently introduced feed ingredients such as MM, RM and BC was detailed in Supporting Information File 1.

### 2.5 Determination of Production Performance

#### 2.5.1 BWG and Feed Intake (FI)

The hen was weighed at the beginning of the experiment (Initial body weight; IBW) and again at the end of it (final body weight; FBW). The difference between the initial and the ultimate body weight was used to compute body weight gain (BWG) or decrease. The amount of feed supplied and feed rejection in each cage (replicate) were weighed each week to calculate the amount of feed consumed. The amount of feed consumed each replication was determined by subtracting the weight of the feed that was refused (leftover feed) from the total amount of feed that was administered (Hossain et al. 2025).

$$\mathrm{Body}\;\mathrm{weight}\;\mathrm{gain}\left(\mathrm{BWG}\right)=\mathrm{FBW}-\mathrm{IBW}$$

$$\mathrm{Feed}\;\mathrm{intake}\left(\mathrm{FI}\right)=\mathrm{Feed}\;\mathrm{offered}-\mathrm{Feed}\;\mathrm{refusal}$$

#### 2.5.2 Hen Day Egg Production Per cent

The precise formula was used to obtain the replication‐wise hen day egg production percentage.

$$\begin{array}{ccc} & & \mathrm{Hen}\;\mathrm{day}\;\mathrm{egg}\;\mathrm{production}\;\left(\mathrm{HDEP}\;\%\right)\\ & & \;=\;\frac{\mathrm{No}.\;\mathrm{of}\;\mathrm{eggs}\;\mathrm{laid}\;\times \;\mathrm{no}\;\mathrm{of}\;\mathrm{hen}\;}{\mathrm{Total}\;\mathrm{no}.\;\mathrm{of}\;\mathrm{days}}\times 100 \end{array}$$

#### 2.5.3 Egg Weight

Prior to quality assessment, egg weight was measured using a digital balance (Shimadzu Semi‐Micro Analytical Balance, AP225WD Dual Range; Japan).

### 2.6 Determination of Egg Quality

During the eighth and sixteenth weeks of the experimental period, nine eggs from each replication of the six treatments were taken into consideration to assess the characteristics of the egg quality. Each egg was washed with a moist towel before being promptly collected and numbered using wooden pencils in accordance with replication in order to determine its quality.

#### 2.6.1 Egg Shape Index, Albumen Index and Yolk Index

The ratio of an egg's largest width to its maximum length is called the egg shape index. The following method was used to calculate the egg shape index for each egg.

$$\mathrm{Egg}\;\mathrm{shape}\;\mathrm{index}=\frac{\mathrm{Width}\;\mathrm{of}\;\mathrm{the}\;\mathrm{egg}}{\mathrm{Length}\;\mathrm{of}\;\mathrm{egg}}\times 100$$

In each instance, the average of nine measurements was used to determine breadth and length. The following formula was used to calculate the albumen and yolk index (Wang et al. 2019).

$$\mathrm{Albumen}\;\mathrm{index}=\frac{\mathrm{Average}\;\mathrm{height}\;\mathrm{of}\;\mathrm{thick}\;\mathrm{albumen}}{\mathrm{Average}\;\mathrm{diameter}\;\mathrm{of}\;\mathrm{thick}\;\mathrm{albumen}}$$

$$\mathrm{Yolk}\;\mathrm{index}=\frac{\mathrm{Average}\;\mathrm{height}\;\mathrm{of}\;\mathrm{yolk}}{\mathrm{Average}\;\mathrm{diameter}\;\mathrm{of}\;\mathrm{yolk}}$$

The height of the yolk was measured by a spherometer (Double Disc; India) and the diameter by slide calipers (HengLiang Digital Slide Caliper; China).

#### 2.6.2 Determination of Haugh Unit

Egg's Haugh unit (HU) was determined based on the formula of Narushin et al. (2021).

$$\mathrm{HU}=100\log\left(H+7.57-1.7W^{0.17}\right)$$

where HU is the Haugh unit, *H* is the Height of thick albumen and *W* is the Egg weight (g).

#### 2.6.3 Shell Thickness and Per cent of Shell

The egg shell was immediately broken, steeped in lukewarm water for about 2 h, and then the shell‐membranes were removed and then the thickness (mm) of the shell was determined using screw gauze. Each shell had three measurements taken from it at three separate points: twice from the waist and once from each end. After measuring the shell then it was dried in hot air oven and weight in digital balance. Following weighing, the overall egg shell weight was determined and the percentage of shell was calculated.

#### 2.6.4 Microbial Sampling and Count Gut Morphology

At the end of the trial (16 weeks), faeces was collected from three chickens per replicate. The faeces was utilized for a subsequent serial dilution study of the bacterial colony. Serial dilutions of 1 g of faeces (1:10) to 10–6 with a 0.85 sodium chloride (NaCl) solution were performed. The total bacterial count (TBC) and *Escherichia coli* counts were then cultured using 0.1 mL of each dilution on Plate Count Agar and EMB agar, respectively.

### 2.7 Statistical Analyses

All collected data from each treatment group were systematically recorded in an Excel spreadsheet for organization and preliminary review (MS Excel 2013). The statistical analyses were performed using IBM SPSS Statistics (Version 26.0) to ensure accurate and reliable interpretation of the experimental results. A one‐way analysis of variance (ANOVA) was used to evaluate the effects of different treatments on the measured parameters. This method was chosen as it allows comparison of means across multiple independent groups. Following the ANOVA, Duncan's Multiple Range Test (DMRT) and Tukey's Honest Significant Difference (HSD) test were applied as post hoc tests to identify specific differences between treatment groups. DMRT was used due to its ability to detect variations among groups with better precision, whereas Tukey's HSD was applied to control the family‐wise error rate when making multiple comparisons. The statistical significance was determined at *p *< 0.05, meaning any difference observed with a probability value below this threshold was considered statistically significant. All results were expressed as mean ± standard error of the mean (SEM) to indicate variability within the data.

## 3 Results

### 3.1 Production Performance of Laying Hens

The production performance parameters, including body weight, feed intake, egg weight and hen‐day egg production, of laying hens fed different dietary protein sources are presented in Table 2.

**TABLE 2:** Production performance of laying hens fed different protein source ingredients on laying hen during the experimental period (36–52 weeks of age).

Performance metrics	Age periods (Weeks)	Dietary treatment						Level of sig.
		T0	T1	T2	T3	T4	T5	
Body weight (g)	Initial weight (36th)	1933.00 ± 55	1914.33 ± 14.85	1864.40 ± 46.79	1845.53 ± 38.35	1995.53 ± 22.79	1831.63 ± 41.12	NS
	Final weight (52nd)	2013.33 ± 21.67^b^, ^c^	1964.0 ± 34.90^a^, ^b^, ^c^	1924.67 ± 30.70^a^, ^b^	1895.67 ± 45.37^a^	2039.0 ± 18.01^c^	1928.0 ± 29.02^a^, ^b^	^a^
Feed intake (g/bird/day)	1st month (37–40 weeks)	115.50 ± 0.50	116.08 ± 0.04	114.67 ± 0.60	115.58 ± 1.02	115.53 ± 0.16	114.83 ± 0.96	NS
	2nd month (41–44 weeks)	112.92 ± 0.51	113.50 ± 1.09	114.67 ± 0.96	114.67 ± 0.96	114.50 ± 0.50	114.50 ± 1.13	NS
	3rd month (45–48 weeks)	115.92 ± 0.08	114.25 ± 0.00	116.08 ± 0.74	115.08 ± 1.12	115.08 ± 0.30	114.08 ± 0.60	NS
	4th month (49–52 weeks)	115.58 ± 0.79	115.75 ± 0.66	115.0 ± 0.76	114.75 ± 0.14	115.50 ± 0.52	115.83 ± 0.51	NS
	Average	114.90 ± 0.27	114.73 ± 0.17	115.16 ± 0.31	114.89 ± 0.50	115.07 ± 0.35	114.81 ± 0.11	NS
Egg weight (g)	1st month (37–40 weeks)	63.08 ± 0.37	62.32 ± 0.59	61.71 ± 0.96	62.22 ± 0.78	62.99 ± 0.71	62.53 ± 0.89	NS
	2nd month (41–44 weeks)	59.44 ± 0.26	61.76 ± 1.33	60.49 ± 1.06	60.63 ± 0.12	62.65 ± 0.79	61.13 ± 1.47	NS
	3rd month (45–48 weeks)	60.15 ± 0.50^a^	62.54 ± 0.09^b^	61.06 ± 0.33^a^, ^b^	63.05 ± 0.90^b^	63.65 ± 0.81^b^	61.24 ± 0.62^a^, ^b^	^a^
	4th month (49–52 weeks)	59.96 ± 0.61	62.26 ± 0.88	59.68 ± 0.61	61.14 ± 0.48	60.67 ± 0.40	61.19 ± 0.38	NS
	Average	60.66 ± 0.12	62.22 ± 0.70	60.73 ± 0.23	61.76 ± 0.30	62.24 ± 0.89	61.52 ± 0.71	NS
% Hen‐day egg production	1st month (37–40 weeks)	91.06 ± .34	86.84 ± 1.66	91.57 ± 0.44	88.86 ± 0.87	89.95 ± 0.64	89.48 ± 1.30	NS
	2nd month (41–44 weeks)	88.29 ± 0.20	86.31 ± 1.03	87.89 ± 1.30	86.90 ± 0.003	87.30 ± 1.59	86.50 ± 2.10	NS
	3rd month (45–48 weeks)	88.29 ± 0.52	86.90 ± 0.00	88.09 ± 1.03	87.30 ± 0.20	87.70 ± 0.86	88.29 ± 0.78	NS
	4th month (49–52 weeks)	88.09 ± 0.34	86.74 ± 0.42	89.29 ± 0.64	88.06 ± 1.16	88.89 ± 0.40	88.29 ± 0.40	NS
	Average	88.93 ± 0.28	86.74 ± 0.42	89.20 ± 0.80	87.77 ± 0.52	88.46 ± 0.13	88.29 ± 0.78	NS

*Note*: The mean values with different superscripts (a–c) within the same row differ significantly, at least (*p* < 0.05). All values indicate mean ± standard error of mean. NS means statistically not significant. The mean was calculated from 36 chickens out of 54 in each treatment group.

Abbreviation: NS, non‐significant.

^a^ Means significant at 5% level of significance (*p* < 0.05).

^b^ *p* < 0.01.

^c^ *p* < 0.001.

#### 3.1.1 Body Weight (BWT)

The IBW of the hens at 36 weeks of age did not differ significantly among the treatment groups (*p *> 0.05). However, at 52 weeks, significant differences (*p *< 0.05) were observed in FBW among the groups. The highest FBW was recorded in the T4 group (2039.0 ± 18.01 g), followed by T0 (2013.33 ± 21.67 g), whereas the lowest was found in T3 (1895.67 ± 45.37 g). The T5 group, which included rapeseed meal and BC, also showed a moderate FBW (1928.0 ± 29.02 g).

#### 3.1.2 Feed Intake (FI)

Throughout the experimental period (37–52 weeks), feed intake did not significantly vary among the dietary treatment groups (*p *> 0.05). The daily feed intake ranged from 112.92 ± 0.51 g to 116.08 ± 0.04 g per bird across different treatment groups, with no notable differences observed in the first, second, third or fourth months. The average feed intake remained relatively stable across all groups, suggesting that the inclusion of MM, rapeseed meal and BC did not negatively impact feed consumption.

#### 3.1.3 Egg Weight

Egg weight remained statistically non‐significant (*p *> 0.05) in most of the study periods, except for the third month (45–48 weeks), where significant differences (*p *< 0.05) were noted among treatments. The highest egg weight in this period was observed in the T4 group (63.65 ± 0.81 g), followed by T3 (63.05 ± 0.90 g) and T1 (62.54 ± 0.09 g), whereas the lowest was recorded in T2 (61.06 ± 0.33 g). However, overall, the average egg weight across the entire trial period did not differ significantly among the groups (*p *> 0.05).

#### 3.1.4 Hen‐Day Egg Production (HDEP %)

The percentage of hen‐day egg production remained statistically non‐significant (*p *> 0.05) across all treatment groups throughout the experiment. The highest average hen‐day egg production was recorded in the control group (T0: 88.93% ± 0.28%), whereas the lowest was observed in the T1 group (86.74% ± 0.42%). However, no significant variations were found between treatment groups during the 4‐month trial period, indicating that the inclusion of MM, rapeseed meal and BC in the diet did not adversely affect egg production.

### 3.2 Egg Quality Characteristics of Laying Hens Fed Different Source Feed Ingredients

The results of egg quality characteristics from hens fed different dietary treatments during the experimental period (36–52 weeks of age) are summarized in Table 3.

**TABLE 3:** Egg quality characteristics of laying hen fed different source feed ingredients during the experimental period (36–52 weeks of age).

Egg quality characters	Period (age) (week)	Dietary treatment						Level of sig.
		T0	T1	T2	T3	T4	T5	
Per cent shell	44	10.80 ± 0.12	10.54 ± 0.06	10.19 ± 0.36	10.18 ± 0.11	10.14 ± 0.28	10.65 ± 0.19	NS
	52	10.46 ± 0.08	9.88 ± 0.43	10.45 ± 0.08	10.60 ± 0.60	10.56 ± 0.08	10.69 ± 0.04	NS
Shell thickness (mm)	44	0.38 ± 0.02	0.39 ± 0.01	0.37 ± 0.02	0.36 ± 0.04	0.38 ± 0.02	0.37 ± 0.02	NS
	52	0.37 ± 0.03	0.38 ± 0.00	0.39 ± 0.0	0.38 ± 0.02	0.36 ± 0.01	0.39 ± 0.01	NS
Shape index	44	76.66 ± 0.38	75.59 ± 0.80	76.22 ± 0.16	76.72 ± 0.86	77.17 ± 0.65	77.45 ± 0.45	NS
	52	77.01 ± 0.30	75.98 ± 1.22	76.28 ± 0.89	76.04 ± 0.56	76.54 ± 0.01	76.96 ± 0.78	NS
Albumin index	44	0.11 ± 0.00	0.11 ± 0.00	0.11 ± 0.00	0.11 ± 0.00	0.11 ± 0.00	0.11 ± 0.00	NS
	52	0.10 ± 0.01	0.10 ± 0.0	0.11 ± 0.00	0.11 ± 0.01	0.11 ± 0.00	0.11 ± 0.00	NS
Haugh unit	44	83.46 ± 0.56	83.50 ± 1.23	81.33 ± 0.35	83.89 ± 0.97	83.67 ± 1.23	81.84 ± 0.15	NS
	52	82.55 ± 0.61	81.50 ± 0.30	82.96 ± 0.91	81.90 ± 0.80	83.60 ± 1.00	83.76 ± 0.59	NS
Yolk index	44	0.41 ± 0.01	0.42 ± 0.01	0.41 ± 0.01	0.41 ± 0.01	0.41 ± 0.01	0.43 ± 0.01	NS
	52	0.44 ± 0.0^c^	0.42 ± 0.2^bc^	0.39 ± 0.00^a^	0.41 ± 0.01^abc^	0.39 ± 0.00^a^	0.42 ± 0.01^bc^	^a^
Shell breaking strength	44	2.25 ± 0.08	2.14 ± 0.03	2.22 ± 0.08	2.41 ± 0.12	2.21 ± 0.01	2.20 ± 0.01	NS
	52	2.16 ± 0.02	2.16 ± 0.02	2.15 ± 0.04	2.21 ± 0.08	2.23 ± 0.07	2.31 ± 0.01	NS

*Note*: The mean values with different superscripts (a–c) within the same row differ significantly, at least (*p *< 0.05). All values indicate mean ± standard error of mean. NS means statistically not significant.

^a^ Means significant at 5% level of significance (*p *< 0.05).

#### 3.2.1 Shell Quality

The percentage of eggshell did not significantly differ (*p *> 0.05) among dietary treatments at both the 44th and 52nd weeks of age. Shell thickness also showed no significant variation across treatments, with values ranging from 0.36 to 0.39 mm at both time points. Similarly, shell breaking strength remained statistically non‐significant among treatments, with values between 2.14 and 2.41 kg/cm^2^ at 44 weeks and 2.15 and 2.31 kg/cm^2^ at 52 weeks.

#### 3.2.2 Egg Shape and Internal Quality

The shape index, which is an indicator of egg shape, showed no significant differences among treatments at either the 44th or 52nd week. Values ranged from 75.59 to 77.45 at 44 weeks and 75.98 to 77.01 at 52 weeks, indicating uniformity in egg shape.

The albumin index remained consistent across treatments, with no significant variation observed at either time point (*p *> 0.05). Similarly, the HU, which is a critical indicator of egg freshness and albumin quality, did not show significant differences among treatments. The HU values ranged from 81.33 to 83.89 at 44 weeks and 81.50 to 83.76 at 52 weeks, suggesting that the dietary treatments had no substantial effect on albumen quality.

#### 3.2.3 Yolk Index

The yolk index was the only parameter that showed a significant difference (*p *< 0.05) among treatments at 52 weeks of age. Treatment T2 (0.39 ± 0.00) and T4 (0.39 ± 0.00) exhibited significantly lower yolk index values compared to T0 (0.44 ± 0.00) and T5 (0.42 ± 0.01). However, no significant differences were observed at 44 weeks, where values ranged between 0.41 and 0.43 across all groups.

### 3.3 Effect on Faecal *E. coli* and Production Economics in Laying Hens

#### 3.3.1 Faecal *E. coli* and Gut Microflora Count

The faecal *E. coli* and gut microflora count at the end of the trial (52nd week) showed significant differences (*p *< 0.05) among the dietary treatments (Tables 4 and 5). The highest *E. coli* colony count was observed in the control group (T0: 229.67 ± 8.35), which was significantly higher than all other treatments. T1 (212.33 ± 6.49) had a slightly lower count but was still significantly higher than T2, T3, T4 and T5. Among the experimental groups, T3 (144.00 ± 12.42) and T5 (145.00 ± 8.89) had the lowest bacterial counts, which were significantly different from the control and other groups. T2 (185.00 ± 3.46) and T4 (168.33 ± 7.06) showed intermediate values.

**TABLE 4:** Effect of dietary different protein source feed ingredients on gut microflora of laying hens at the end of trial (52nd week).

	Dietary treatment (CFU × 10^10^)						Level of significance
Age	T0	T1	T2	T3	T4	T5	
At 52 weeks of age	410 ± 5.77^c^ × 10^10^	376.67 ± 24.04^bc^ × 10^10^	380.00 ± 5.77^bc^ × 10^10^	380.00 ± 11.55^bc^ × 10^10^	350.00 ± 20.82^ab^ × 10^10^	320.00 ± 5.77^a^ × 10^10^	^a^

*Note*: The mean values with different superscripts (a–c) within the same row differ significantly, at least (*p* < 0.05). All values indicate mean ± standard error of mean. NS means statistically not significant.

^a^ Means significant at 5% level of significance (*p* < 0.05).

**TABLE 5:** Effect of dietary different protein source feed ingredients on faecal *Escherichia coli* of laying hens at the end of trial (52nd week).

Treatments	Bacterial colony on EMB (mean ± SE)	*F* value	*p* value
T0	229.67 ± 8.35^a^	18.25	0.0001
T1	212.33 ± 6.49^ab^		
T2	185.00 ± 3.46^bc^		
T3	144.00 ± 12.42^d^		
T4	168.33 ± 7.06^cd^		
T5	145.00 ± 8.89^d^		

*Note*: Values are mean ± SE, one‐way ANOVA (SPSS V‐26, Tukey's HSD). Values with different superscripts latters ‘a–d’ in the same row differ significantly (*p* < 0.05), 5% level of significance.

Abbreviation: SE, standard error.

#### 3.3.2 Economic Analysis of Feed and Egg Production

The cost of feed per kilogram varied across dietary treatments (Table 6). The highest feed cost was observed in the control group (T0: 43.7 Tk./kg), followed by *T*_1_ (43.0 Tk./kg). The lowest feed cost was found in T4 (39.5 Tk./kg), whereas other treatments ranged between 40.0 and 40.5 Tk./kg.

**TABLE 6:** Effect of different protein source feed on feed cost and per egg production.

Variables	Dietary treatments					
	T0	T1	T2	T3	T4	T5
Feed cost/kg mixed feed (Tk.)	43.7	43.0	40.0	40.5	39.5	40
Production cost/egg	7.66	7.68	7.16	7.30	7.13	7.20

The production cost per egg also varied, with T4 (7.13 Tk./egg) showing the lowest cost, followed by T2 (7.16 Tk./egg) and T5 (7.20 Tk./egg). The highest production cost per egg was recorded in T1 (7.68 Tk./egg), followed by T0 (7.66 Tk./egg). This indicates that alternative protein sources in T2, T3, T4 and T5 could reduce production costs compared to the control and T1 groups.

## 4 Discussion

The findings of this study highlighted the potential of alternative protein sources such as MM, rapeseed meal and BC in laying hen diets as viable substitutes for SBM. The results demonstrated significant improvements in BWG and gut microflora, along with a reduction in feed cost, without negatively affecting feed intake, egg quality or egg production performance.

The inclusion of BC (*N. sativa*), MM and rapeseed meal in poultry diets enhances nutrient utilization and overall performance in laying hens (Azarnik et al. 2010). Black cumin contains bioactive compounds such as thymoquinone, which improve digestive enzyme activity, enhance gut health and promote better nutrient absorption (M. S. Hossain et al. 2024; Navidshad et al. 2025). This leads to improved feed efficiency, increased BWG and enhanced immune function, which collectively support egg production (Aydin et al. 2008). Mustard meal is a rich source of protein and essential amino acids, playing a crucial role in muscle growth and egg formation. It also contains glucosinolates, which, when present in controlled amounts, can stimulate metabolic activity and improve feed utilization (Yılmaz et al. 2024). Additionally, its impact on lipid metabolism contributes to better yolk quality.

Rapeseed meal, another valuable protein source, provides essential amino acids and fibre, which aid in sustained energy release and egg production (Ashnie et al. 2015). However, it contains anti‐nutritional factors such as erucic acid and glucosinolates, which may limit its efficiency unless properly processed (Li et al. 2015). When combined with BC, rapeseed meal demonstrates improved feed conversion and growth performance. Collectively, these alternative protein sources positively influence BWG by improving protein utilization and gut health (Yalçin et al. 2025). They also enhance egg production by optimizing metabolic efficiency and providing essential nutrients. Moreover, their antioxidant and immune‐boosting properties contribute to better overall health, improved egg quality and enhanced feed efficiency in laying hens (Acar et al. 2024).

The inclusion of MM, rapeseed meal and BC positively influenced BWG without significantly affecting feed intake. The highest FBW was observed in the *T*_5_ group (rapeseed meal + BC), followed closely by T4 (MM + BC), indicating that these protein sources enhance nutrient utilization and growth efficiency. In contrast, the moderate BWG in the T3 group (rapeseed meal alone) suggests that rapeseed meal may be more effective when combined with BC.

These findings align with previous studies demonstrating that alternative plant‐based protein sources can support or improve poultry production performance when appropriately formulated (Adegbeye et al. 2020; Akhtar et al. 2003). The superior BWG in the T5 group is consistent with Hossain et al. (2016), who reported that dietary inclusion of BC at levels of 1% or 3% significantly enhanced the FBW of laying hens (*p *< 0.05). However, Hossain et al. (2016) also noted that BC inclusion at 1%, 1.5% or 2% had no significant impact on body weight, highlighting the need for optimal dietary formulation to maximize growth benefits.

The absence of significant differences in feed intake across dietary treatments suggests that replacing SBM with MM, rapeseed meal and BC does not affect the palatability or acceptability of the feed. This is an important finding for commercial poultry nutrition, as any negative impact on feed intake could reduce production efficiency.

Egg weight remained largely unaffected by dietary treatments, with a significant difference observed only in the third month of the trial. The highest egg weight recorded in the T4 group may be attributed to the synergistic effects of MM and BC, which likely enhanced nutrient assimilation and metabolism. However, the overall non‐significant differences across treatments indicate that alternative protein sources do not compromise egg size, a critical economic trait in commercial egg production.

Diets containing rapeseed meal (RM) and BC showed no significant effect on average egg weight, consistent with previous studies. Hossain et al. (2016) reported that BC supplementation at 1%, 1.5%, 2.0% and 0% had no impact on egg weight in laying hens. Similarly, Gheisari and Ghayor (2014) found no adverse effects when replacing 15%–20% of SBM with RM, and Ciurescu (2023) noted no significant variations in egg weight with up to 52.38% RM substitution for SBM. Conversely, some studies have reported increased egg weight with BC supplementation. Aydin et al. (2008) observed that BC at 2%–3% levels increased egg weight, whereas Khan et al. (2013) found a positive impact at 4%–5% inclusion. However, Tan et al. (2022) suggested that feeding laying ducks a 10% RM diet reduced egg weight.

The lack of significant changes in egg weight with MM and RM inclusion may be due to their balanced nutrient profile, which meets the protein and amino acid requirements of laying hens without adversely affecting egg size. Additionally, proper processing of these ingredients likely reduced the presence of anti‐nutritional factors, ensuring optimal nutrient utilization.

In our study, replacing SBM with rapeseed meal, MM and BC had no negative impact on egg quality parameters, including eggshell properties, shape index, albumen index and HU. Hossain et al. (2016) reported that BC supplementation had no significant effect on egg weight or other egg quality parameters, including HU, colour score, albumen index, yolk index and shell thickness (*p *> 0.05), though yolk index was significant (*p *< 0.05) at 52 weeks of age. This contradicts with Akhtar et al. (2003), who found no significant (*p *> 0.05) change in yolk index with 1.5% BC supplementation. Ciurescu (2023) observed a decline in egg weight with rapeseed meal inclusion up to 15%, but yolk weight remained unaffected. The highest HU in our study was recorded in T4 (MM with BC) and T5 (rapeseed meal with BC), aligning with Gheisari and Ghayor (2014), who found that replacing 15%–20% of SBM with RM increased HU without negatively affecting egg weight, yolk weight or yolk colour. Similarly, Wang et al. (2017) reported no adverse effects of rapeseed meal at 148.7 g/kg on egg quality or laying performance, whereas Riyazi et al. (2009) also found no impact of dietary RM on HU.

This can be attributed to the balanced nutrient composition of these alternative protein sources, which provided essential amino acids, minerals and bioactive compounds necessary for maintaining egg quality. Proper processing of rapeseed and MMs likely reduced anti‐nutritional factors such as glucosinolates, ensuring efficient nutrient absorption (Cheva‐Isarakul et al. 2003; Li et al. 2015). Additionally, BC's bioactive compounds may have supported overall metabolic functions, contributing to optimal albumen and yolk quality (Albakry et al. 2022; Pootthachaya et al. 2023). The adequate supply of calcium and phosphorus from these ingredients also helped maintain eggshell strength and integrity (Cheva‐Isarakul et al. 2003).

There was significant (*p *< 0.05) effect of dietary different protein source feed ingredients on gut microflora of laying hens while incorporation of BCs, MM and rapeseed meal. The lowest microbial load found in T4 and T5 groups diet containing 3.5% BC. These results consisted with Boka et al. (2014) indicated that irrespective of the dosage of supplements, BC consumption reduced the number of *E. coli* in the ileal digesta. Additionally, black seed and oil were found to reduce the development of *E. coli*, *S. aureus*, *Brucella melitensis* and total viable bacteria in poultry by Alsawaf and Alnaemi (2011) and Islam et al. (2011).

Black cumin and rapeseed meal help reduce the faecal *E. coli* load in layer chickens through their antimicrobial and gut health‐enhancing properties (Navidshad et al. 2025). Black cumin contains bioactive compounds like thymoquinone, which exhibit strong antibacterial effects by disrupting bacterial cell membranes and inhibiting bacterial enzymes (Abdollahi et al. 2024). Additionally, it promotes beneficial gut microbiota, enhancing competitive exclusion of *E. coli*. Rapeseed meal, on the other hand, contains antimicrobial peptides and glucosinolates, which exert bacteriostatic effects, limiting *E. coli* growth. Proper processing of rapeseed meal reduces anti‐nutritional factors while preserving its bioactive compounds (Konkol et al. 2024). Both ingredients contribute to improved gut integrity and immunity, reducing pathogen colonization and faecal shedding.

This study has some limitations, including the lack of long‐term evaluation of the effects of MM, rapeseed meal and BC on laying performance and immune response. The study did not analyse interactions with other dietary components or assess gut microbiome shifts using advanced sequencing methods. Variations in processing methods for rapeseed and MMs may also influence results. Additionally, environmental factors like biosecurity and hygiene were not extensively considered. Future research should focus on long‐term effects, economic feasibility and microbiome analysis for a comprehensive understanding.

## 5 Conclusions

Mustard meal, rapeseed meal and BC proved to be effective alternatives to SBM in layer diets, maintaining egg production, quality and feed intake without adverse effects. The highest BWG was recorded in T5 (rapeseed meal + BC), whereas egg production remained statistically similar across treatments. Gut microflora analysis showed significantly lower *E. coli* counts in T5, indicating a potential health benefit. Economic analysis revealed that T4 (MM + BC) had the lowest egg production cost, making it the most cost‐effective alternative. These findings highlight the potential of MM, rapeseed meal and BC as sustainable and economical protein sources in commercial layer diets.

## Author Contributions

**Md Abubakar Siddik and Hemayet Hossain**: conceptualization, methodology, investigation, analysis, software, writing – original draft, writing – reviewing and editing. **Shoriful Islam, Md. Azizul Haque, Sweety Rani Mondal and Khadiza Akter Brishty**: methodology, investigation, software, analysis, writing – reviewing and editing. **Mst Afroza Khatun and Md. Mahfujur Rahman**: supervision, validity, project administration, fund acquisition, writing – original draft, writing – reviewing and editing.

## Conflicts of Interest

The authors declare no conflicts of interest.

## Supporting information




**Supporting File 1**: Nutrient composition of raw Mustard Meal, Rapeseed Meal and Black cumins.

## Data Availability

Data are available upon request from the corresponding author.
