# Supplementary material for: Impact of Soybean Meal, Mustard Meal, Rapeseed Meal and Black Cumin on Production Performance, Egg Quality and Gut Microflora of Laying Hens
Source: Vet Med Sci. 2026 Mar 11;12(2):e70863. doi: 10.1002/vms3.70863 (PMC12978149; doi:10.1002/vms3.70863)
Supplement: Supplementary file 1 — Supporting File 1: Nutrient composition of raw Mustard Meal, Rapeseed Meal and Black cumins. [file VMS3-12-e70863-s001.docx]

**Supporting Information File 1:** Nutrient composition of raw Mustard Meal, Rapeseed Meal and Black cumins.

| Composition (% of Dry matter basis) | Mustard Meal | Rapeseed Meal | Black cumins |
| --- | --- | --- | --- |
| Crude protein (%) | 33 | 37 | 23 |
| Crude fibre (%) | 14 | 11 | 12 |
| Crude fat (%) | 19 | 5 | 34 |
